# Protective Effects of a New Phloretin Derivative against UVB-Induced Damage in Skin Cell Model and Human Volunteers

**DOI:** 10.3390/ijms151018919

**Published:** 2014-10-20

**Authors:** Seoungwoo Shin, Hyunwoo Kum, Dehun Ryu, Minkyung Kim, Eunsun Jung, Deokhoon Park

**Affiliations:** Biospectrum Life Science Institute, Seoungnam City, Gyunggi Do 462-807, Korea; E-Mails: biost@biospectrum.com (S.S.); biosd@biospectrum.com (H.K.); biosc@biospectrum.com (D.R.); biotq@biospectrum.com (M.K.)

**Keywords:** phloretin 3',3-disulfonate, photoprotection, anti-inflammation, sulfonation

## Abstract

The phenolic compound phloretin is a prominent member of the chemical class of dihydrochalcones. Phloretin is specifically found in apple and apple juice and known for its biological properties. We were particularly interested in its potential dermo-cosmetic applications. However, practical limitations of phloretin do exist due to its poor water-solubility. Phloretin was sulfonated with sulfuric acid (98%, wt) and mixed with saturated salt water to produce phloretin 3',3-disulfonate in order to increase its water-solubility. Here we reported the photoprotective effect of phloretin 3',3-disulfonate (PS), a new semi-synthetic derivative of phloretin. Results showed that PS attenuated cyclobutane pyrimidine dimer (CPDs) formation, glutathione (GSH) depletion and apoptosis induced by ultraviolet B (UVB). The photoprotective effect of PS is tightly correlated to the enhancement of nucleotide excision repair (NER) gene expression. Furthemore, PS had inhibitory effects on UVB-induced release of the inflammatory mediators, such as IL-6 and prostaglandin-E_2_. We also confirmed the safety and clinical efficacy of PS on human skin. Overall, the results demonstrated significant benefits of PS on the protection of keratinocytes against UVB-induced injuries and suggested its potential use in skin photoprotection.

## 1. Introduction

Solar ultraviolet (UV) light can penetrate the atmosphere and cause most known skin disorders [1,2]. Acute UV irradiation can elicit various responses including sunburn, inflammation, DNA damage, and apoptosis [3,4,5]. Chronic and repetitive UV irradiation can lead to photo-aging, sustained immune suppression, and carcinogenesis of the skin [3,6].

The UVB range of solar radiation can penetrate the epidermis of the skin, inducing both direct and indirect DNA damaging effects. UV radiation depletes the cutaneous defense system and leads to the accumulation of DNA damage, excessive cell apoptosis, skin aging, and impaired epidermal integrity [7,8].

There has been considerable interest in the use of naturally occurring botanicals for the protection of human skin from UV-induced damage. Flavonoids and other phenolic compounds have been considered as a major class of protectants against UV-induced damage [9,10,11]. However, phenolic compounds application is restricted due to several factors including diversified potency, molecule toxicity, difficult purification strategies or insolubility in water. Much better solubility is shown by some sulfonic derivatives of phenolic compounds, which at the same time retain the properties of the parent compound [12,13,14].

**Figure 1:**
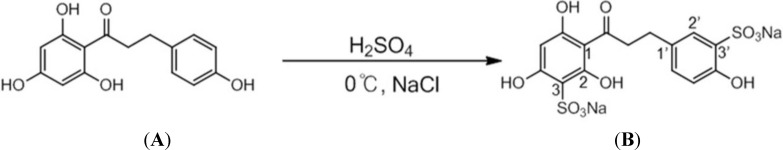
Synthesis reaction from phloretin (**A**) to produce phloretin 3',3-disulfonate (**B**).

Phloretin is known for its biological properties such as antioxidative [15,16], antimicrobial [17], antitumor [18,19], and anti-inflammatory activity [20,21]. However, there are practical limitations to the use of phloretin due to its poor water-solubility. It has been widely accepted that sulfonation could enhance water solubility. We synthesized a soluble derivative of phloretin via the sulfonation process (Figure 1). The phloretin derivative, phloretin 3',3-disulfonate is characterized by good aqueous solubility and low toxicity. Therefore, phloretin 3',3-disulfonate is potentially useful for therapy.

Our studies have focused more on the photo-protective effect of the soluble derivative of phloretin than on the photo-protective effect of the parent compounds. We investigated the protective effects of phloretin 3',3-disulfonate on UV-induced damage in keratinocytes in this study. We also evaluated skin recovery and safety after topical application of phloretin 3',3-disulfonate in humans, to determine the feasibility of its clinical use.

## 2. Results and Discussion

### 2.1. Analysis of Phloretin and Phloretin 3',3-Disulfonate

**Figure 2:**
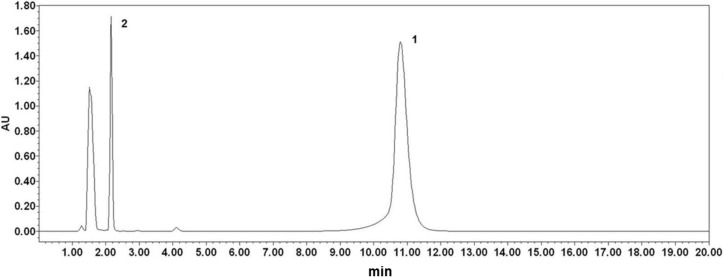
The profile of phloretin (**1**) and phloretin 3',3-disulfonate (**2**) by HPLC.

A typical HPLC chromatogram of phloretin and phloretin 3',3-disulfonate was shown in Figure 2. The results showed no overlap with dihydrochalcones at 285 nm. The HPLC analysis showed that phloretin was eluted at 10.797 min and phloretin 3',3-disulfonate was eluted at 2.165 min. This result indicated that the changes of the polarity were found to be in the order of phloretin 3',3-disulfonate > phloretin.

### 2.2. Solubilities of Phloretin and Phloretin 3',3-Disulfonate

The solubility of phloretin 3',3-disulfonate was determined by comparison with that of phloretin. The solubility of phloretin was 7 μg/mL, while phloretin sodium sulfonation was 187,292 μg/mL (Table 1). The solubility of phloretin and phloretin 3',3-disulfonate showed that a sodium sulfonation moiety could greatly enhance the water solubility of the original compound.

**Table 1:** Solubility of phloretin and phloretin sodium sulfonation.

No.	Test Material	Solubility in Water (μg/mL) ^a^	Relative Solubility
1	Phloretin	7 ± 1	1
2	Phloretin 3',3-disulfonate	187,292 ± 940	26,756

^a^ Mean ± standard deviation (*n* = 3).

### 2.3. Phloretin 3',3-Disulfonate Inhibits UV-Induced Damage in HaCaT Cells

**Figure 3:**
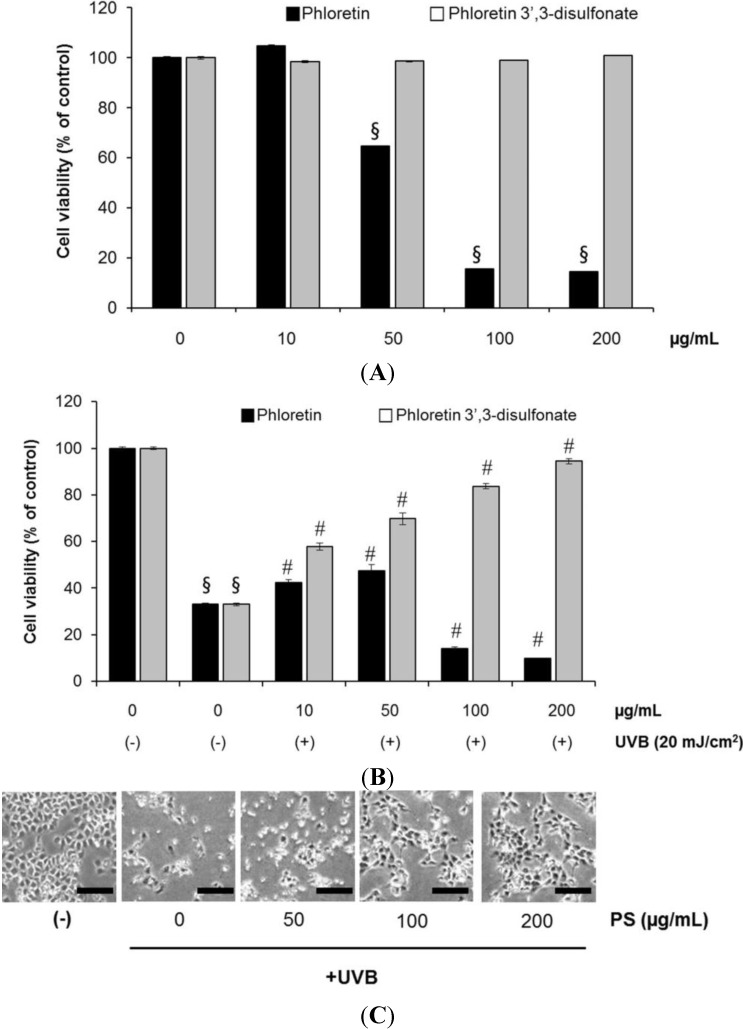
Phloretin 3',3-disulfonate treatment protects HaCaT cells against UVB-mediated phototoxicity. HaCaT cells were treated with phloretin or phloretin 3',3-disulfonate at the same indicated concentration for 12 h after being exposed to UVB, to compare the effects of phloretin and phloretin 3',3-disulfonate. (**A**) MTT assay was used to evaluate cell viability post-treatment with phloretin and phloretin 3',3-disulfonate; (**B**) The percentage of viable cells was assessed by the MTT assay at 12 h after UVB irradiation and (**C**) Phase contrast microscopy. HaCaT cells were irradiated with UVB (20 mJ/cm^2^) and then incubated without or with phloretin 3',3-disulfonate (50–200 µg/mL) for 12 h. Scar bar = 200 µm. A representative picture from 3 independent experiments with similar result is shown. ^§^ *p* < 0.01 compared with the vehicle-treated group, ^#^ *p* < 0.01 compared with the UVB treated group; (*N* = 5); PS: Phloretin 3',3-disulfonate.

We initially investigated the effect of phloretin 3',3-disulfonate (50–200 µg/mL) on the UVB-mediated decrease in cell viability. As expected, UVB (20 mJ/cm^2^) irradiation of HaCaT cells resulted in decreased cell viability. UVB-induced cell growth inhibition was significantly attenuated by treatment with phloretin 3',3-disulfonate (50–200 µg/mL) in a concentration-dependent manner, as determined by the MTT assays (Figure 3A). Phloretin 3',3-disulfonate alone (w/o UVB) at the treated concentration, did not show any significant effects on cell viability. The cytotoxic activity of phloretin 3',3-disulfonate was markedly reduced compared with the parent compounds (Figure 3A). Comparison of the protective effects of phloretin 3',3-disulfonate with those of phloretin, the parent compound, demonstrated comparable efficiency of phloretin 3',3-disulfonate in the prevention of UVB-induced cell death. Phloretin 3',3-disulfonate showed a dose-dependent cytoprotective effect, whereas phloretin was less effective (Figure 3B).

**Figure 4:**
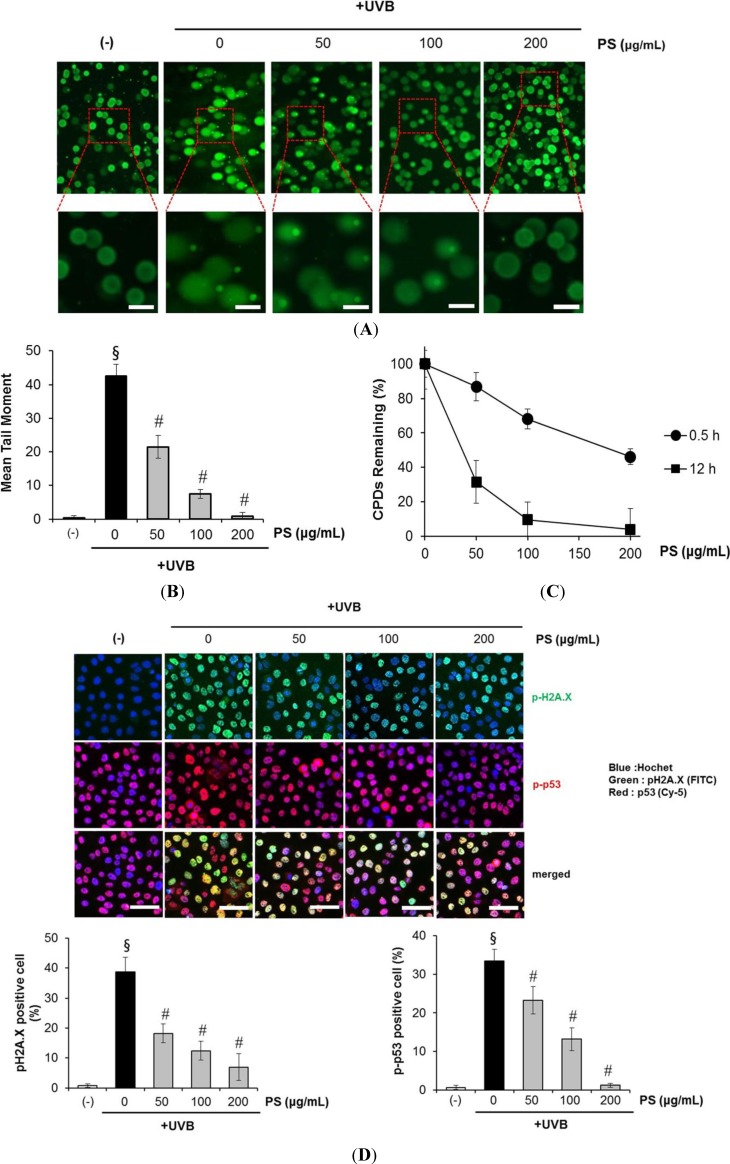
Effect of phloretin 3',3-disulfonate on UVB-induced DNA damage. Cells were treated with different concentrations of phloretin 3',3-disulfonate (50–200 μg/mL) for 12 h after being exposed to UVB (20 mJ/cm^2^). (**A**) At 12 h after UVB irradiation, the cellular DNA damage was detected by the comet assay after rewinding the DNA with alkaline buffer. Slides were stained with SYBR and viewed under a fluorescence microscope. Scar bar = 40 µm; (**B**) Tail lengths (µm) of a minimum of 50 comets in each sample were analyzed using software image analysis; (**C**) Enzyme-linked immunosorbent assay (ELISA) analysis of the percentage (%) of cyclobutane pyrimidine dimers (CPD) remaining in cells pretreated with vehicle or different concentrations of phloretin 3',3-disulfonate (50–200 µg/mL) at 30 min before UVB irradiation (20 mJ/cm^2^) and (**D**) Cells (2 × 10^4^ cell/100 μL) were prepared in 96 well culture plates and fixed/stained to measure the levels of phosphoactive histone H2A.X (γH2A.X; green) and phospho-p53 (serine 15) (red). Staining with Hoechst 33342 (blue) was performed to observe cell nuclei. Cells were imaged on the GE IN Cell Analyzer 1000 at 20× objective magnification. Scar bar = 200 µm. Data are mean ± standard deviation of 3 independent experiments.^§^ *p* < 0.01 compared with the vehicle-treated group, ^#^ *p* < 0.01 compared with the UVB treated group. (*N* = 50); PS: Phloretin 3',3-disulfonate.

We then determined and verified the photoprotective effect of phloretin 3',3-disulfonate on UVB-induced cellular DNA damage using the comet assay. As shown in Figure 4A,B, exposure of HaCaT cells to UVB radiation (20 mJ/cm^2^) resulted in extensive DNA damage, as reflected by the comet tail length, compared to cells that were not exposed to UVB radiation. However, treating cells with phloretin 3',3-disulfonate (50–200 µg/mL) resulted in a reduced amount of DNA damage compared to untreated cells exposed to UVB, as evidenced by the comet structure.

UVB caused direct DNA damage results from the absorption of high energy photons by cellular DNA leading to the formation of cyclobutane-pyrimidine dimers (CPDs) and photoproducts, whereas indirect DNA damage results from the generation of reactive oxygen species that facilitate DNA oxidation [1]. CPDs are bulky DNA lesions formed between adjacent pyrimidine nucleotides in the DNA, and if CPDs are not efficiently removed from the genome, they lead to critical mutations, ultimately initiating skin tumors [1,2].

We evaluated the effect of phloretin 3',3-disulfonate on UVB-induced DNA damage in the form of CPDs formation and their repair, to determine whether phloretin 3',3-disulfonate reduced or repaired UVB-induced DNA damage in HaCaT keratinocytes. The number of CPD-positive cells and intensity of staining of CPD-positive cells was markedly decreased in phloretin 3',3-disulfonate-treated cells compared to untreated cells exposed to UVB (Figure 4C), suggesting that phloretin 3',3-disulfonate accelerated the repair of UVB-induced CPDs in HaCaT keratinocytes.

The kinetics of UVB-induced DNA damage was investigated by analyzing phospho-p53 (Ser-15) and γ-H2AX expression levels. UVB irradiation induced p53 (Ser-15) and γ-H2AX phosphorylation in a dose-dependent manner in HaCaT keratinocytes (Figure S1). Additionally, we determined the intracellular location of phospho-p53 (Ser-15) and γ-H2A.X proteins in HaCaT keratinocytes by immunofluorescence staining and confocal microscopy. When the cells were analyzed 12 h after UVB irradiation, the number of Cy5/FITC-positive cells (p-p53, γH2A.X) and intensity of staining of Cy5/FITC-positive cells was markedly decreased in phloretin 3',3-disulfonate -treated cells compared to untreated cells exposed to UVB (Figure 4D). Taken together, these results suggested that phloretin 3',3-disulfonate reduced UVB-induced DNA damage compared to UVB alone-exposed control cells.

### 2.4. Phloretin 3',3-Disulfonate Enhances Expression of the NER Gene in UVB-Irradiated Cells

**Figure 5:**
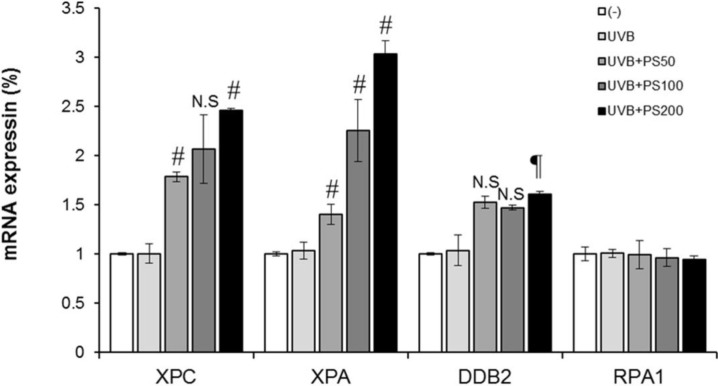
Phloretin 3',3-disulfonate stimulates the mRNA levels of NER genes in UVB-exposed HaCaT keratinocytes. HaCaT cells were exposed to UVB with and without the treatment of phloretin 3',3-disulfonate (50–200 µg/mL). Cells were harvested 3 h later and RNA was extracted. The mRNA levels of NER genes were determined by real-time PCR. NER genes mRNA expression levels are expressed as mean ± SD. Data are mean ± standard deviation of 3 independent experiments. ^¶^ *p* < 0.05 compared with the UVB-treated group, ^#^ *p* < 0.01 compared with the UVB treated group. (*N* = 3); N.S: not significant; PS: Phloretin 3',3-disulfonate.

We found that phloretin 3',3-disulfonate enhanced the removal or repair of UVB-induced DNA damage in UVB-exposed HaCaT keratinocytes, hence we were interested in determining whether the rapid repair or removal of CPDs in UV-exposed skin by phloretin 3',3-disulfonate was mediated via upregulated expression of NER genes. For this purpose, HaCaT keratinocytes were exposed to UVB (20 mJ/cm^2^) with and without the treatment of phloretin 3',3-disulfonate (50–200 μg/mL) in culture media 1 h before UVB exposure. Cells were harvested 3 h after UVB exposure, RNA was isolated and subjected to the analysis of mRNA expression of NER genes (e.g., *XPA*, *XPC*, *DDB2*, and *RPA1*) using real-time PCR. The acute exposure of the HaCaT keratinocyte with UVB slightly enhanced the level of NER genes (not significant) compared to non-UVB-exposed HaCaT keratinocytes. As shown in Figure 5, the mRNA levels of NER genes were significantly enhanced (*p* < 0.05 and *p* < 0.001) in the UVB-exposed HaCaT keratinocytes treated with phloretin 3',3-disulfonate compared to non-UVB-exposed HaCaT keratinocytes. Intriguingly, the enhancement of *XPA* and *XPC* gene levels after the treatment of cells with phloretin 3',3-disulfonate was significantly higher compared to the levels of other NER genes (*DDB2* and *RPA1*). Treatment of HaCaT keratinocytes with phloretin 3',3-disulfonate alone for an identical time period did not significantly induce the levels of NER genes (data not shown). These data suggested that phloretin 3',3-disulfonate might repair UVB-induced DNA damage in HaCaT keratinocytes through the enhancement of the levels of *XPA* and *XPC* genes which have NER properties.

### 2.5. Phloretin 3',3-Disulfonate Protects HaCaT Cells from UVB-Mediated Apoptosis

**Figure 6:**
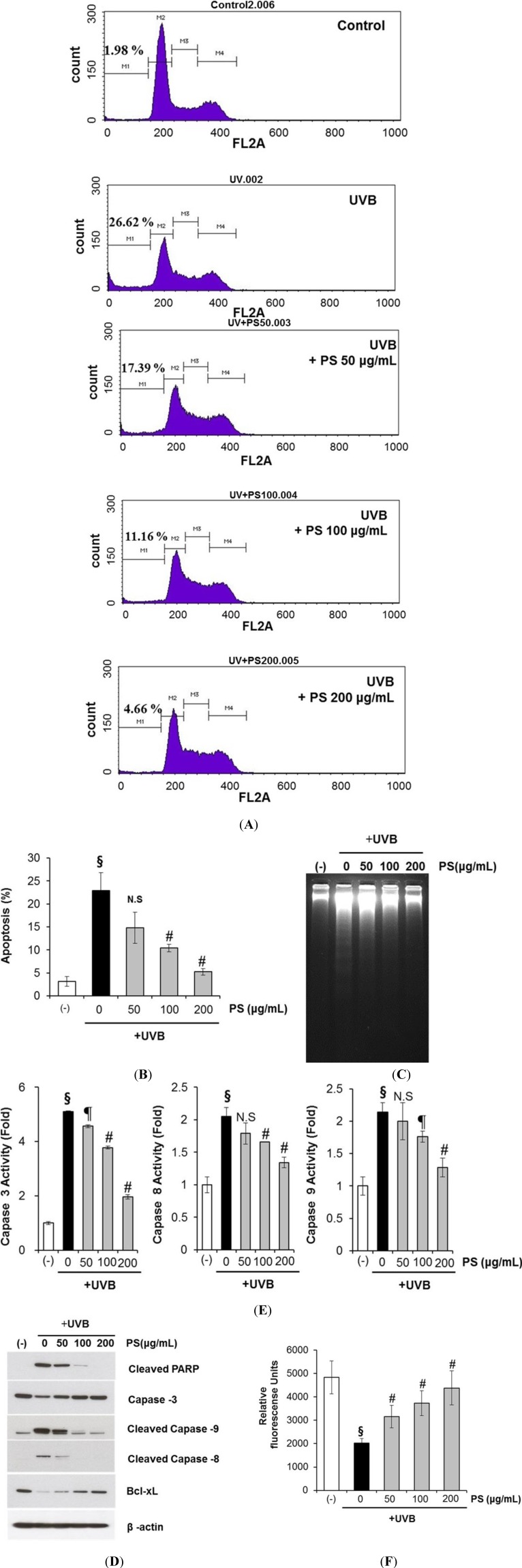
Inhibitory effect of phloretin 3',3-disulfonate on UVB-induced apoptosis. HaCaT cells were treated with phloretin 3',3-disulfonate (50–200 μg/mL) for 12 h after irradiation with 20 mJ/cm^2^ UVB (**A**,**B**) The percentage of cell populations at sub-G1 phase as measured by flow cytometry; (**C**) Agarose gel analysis of fragmentation patterns of cellular DNA isolated from treated or untreated cells; (**D**) Immunoblot analysis of various apoptosis-related proteins in HaCaT cells unexposed or exposed to UVB (20 mJ/cm^2^); (**E**) The activity of caspase-3, -8 and -9 was quantitated colorimetrically and (**F**) Total GSH levels fluorescence units were determined in MCB-loaded cells by using a fluorometer. Data are mean ± standard deviation of 3 independent experiments. ^§^ *p* < 0.01 compared with the vehicle-treated group, ^¶^ *p* < 0.05 compared with the UVB-treated group, ^#^ *p* < 0.01 compared with the UVB treated group. (*N* = 3); N.S: not significant; PS: Phloretin 3',3-disulfonate.

Apoptotic cells were estimated by calculating the number of subdiploid cells in the cell cycle histogram. As shown in Figure 6A,B, a substantial increase in the number of apoptotic cells was observed on exposure to UVB. Phloretin 3',3-disulfonate itself did not induce apoptosis at these concentrations. We found that treating HaCaT cells with phloretin 3',3-disulfonate after UVB irradiation prevented UVB-mediated apoptosis. Next, the UV-triggered apoptosis in HaCaT cells was determined by measuring DNA using agarose gel electrophoresis (Figure 6C). DNA fragmentation decreased significantly in phloretin 3',3-disulfonate-treated cells after exposure to UVB compared to untreated cells. We also evaluated changes in apoptotic marker proteins as a result of UVB irradiation and phloretin 3',3-disulfonate. Previous studies have identified caspases as important apoptosis mediators induced by a range of stimuli [22]. Activation of capase-3, -8 and -9 in HaCaT cells were assessed by immunoblot analysis of lysates from cells were exposed to UVB, with and without phloretin 3',3-disulfonate treatment. Expectedly, we found that UVB irradiation resulted in activated caspase-3, -8 and -9 indicative of cleavage of the proform of caspase into the active form. However, the cleavage was significantly reduced by phloretin 3',3-disulfonate (Figure 6D). UVB irradiation also induced the formation of PARP proteolytic cleavage fragments, indicating imminent apoptosis. The cleaved PARP product markedly increased in untreated cells compared to phloretin 3',3-disulfonate-treated cells upon exposure to UVB (Figure 6D). Taken together, UVB induced procaspase-3 cleavage into the active caspase-3 form, and caspase-3 induced PARP degradation. The relative kinetics of caspase-3, -8 and -9 activation in response to UVB irradiation were measured by colorimetric enzyme assay (Figure 6E). All 3 caspases were activated at 12 h after UVB exposure. Among the caspases tested the effector caspase 3 was activated to the highest extent. Interestingly, a dose-dependent decrease in all 3 caspase activities was found when the UV-irradiated cells were treated with phloretin 3',3-disulfonate (Figure 6E). The results also indicated that phloretin 3',3-disulfonate exhibited a protective effect on UVB-induced apoptosis.

### 2.6. Phloretin 3',3-Disulfonate Prevent UVB-Induced Depletion of GSH in HaCaT Cells

Glutathione (GSH) depletion is a central signaling event that regulates the activation of cell death pathways. GSH depletion is often taken as a marker of oxidative stress and thus, as a consequence of its antioxidant properties scavenging reactive oxygen species (ROS). GSH is well-known antioxidant that is usually present as the most abundant low-molecular-mass thiol in most organisms. It can act as the electron donor for glutathione peroxidase in animal cells, and also directly reacts with ROS [23]. Cellular GSH levels determined with the GSH-sensitive fluorescent dye MCB in HaCaT cells exposed to UVB were significantly decreased. However, the depletion of GSH was significantly protected by the treatment of phloretin 3',3-disulfonate. (Figure 6F). These results indicate that phloretin 3',3-disulfonate is able to regulate the cellular redox signaling events modulating cell death activation and progression by prevention of depleting GSH.

### 2.7. Phloretin 3',3-Disulfonate Inhibits UVB-Induced Inflammation in Vitro and in Vivo

**Figure 7:**
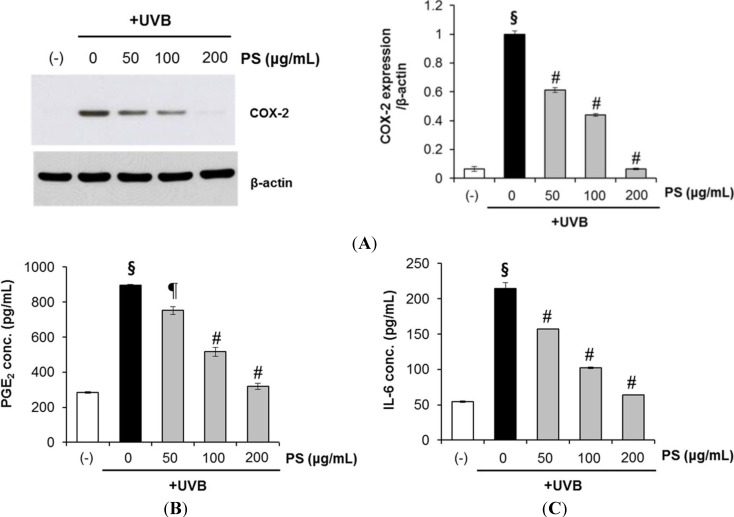
Phloretin 3',3-disulfonate suppresses UVB-mediated inflammation cytokine production in human HaCaT keratinocytes. HaCaT cells were treated with the test substances (50–200 μg/mL phloretin 3',3-disulfonate) after irradiation with 20 mJ/cm^2^ UVB. (**A**) After 12 h, cyclooxygenase (COX)-2 protein expression was assessed by Western blotting. The histogram depicting relative COX-2 protein expression levels was normalized to β-actin expression level; (**B**) Prostaglandin-E_2_ (PGE_2_) and (**C**) interleukin-6 (IL-6) concentrations in cell culture supernatants from the same experiments. Data are mean ± standard deviation of five independent experiments. ^§^ *p* < 0.01 compared with the vehicle-treated group, ^¶^ *p* < 0.05 compared with the UVB-treated group, ^#^ *p* < 0.01 compared with the UVB treated group. (*N* = 3); PS: Phloretin 3',3-disulfonate.

Abnormal upregulation of COX-2 and inflammation play an important role in skin cancer [24]. Studies have shown that UVB irradiation leads to MAPK activation [3] and triggers increased COX-2 expression, which catalyzes the formation of proinflammatory prostaglandins (e.g., PGE_2_) from arachidonic acid [25,26]. Here, we investigated whether phloretin 3',3-disulfonate modulated COX-2 expression in UVB-irradiated HaCaT keratinocytes. As shown in Figure 7A, UVB-induced COX-2 expression clearly decreased following treatment with phloretin 3',3-disulfonate.

The anti-inflammatory effects of phloretin 3',3-disulfonate were further evaluated by analyzing UVB-induced production of pro-inflammatory cytokines such as PGE_2_ and IL-6. UVB (20 mJ/cm^2^) irradiation markedly upregulated PGE_2_ and IL-6, which were suppressed by treatment with phloretin 3',3-disulfonate after UVB irradiation (Figure 7B,C). These results were suggestive of the anti-inflammatory activity of phloretin 3',3-disulfonate.

**Figure 8:**
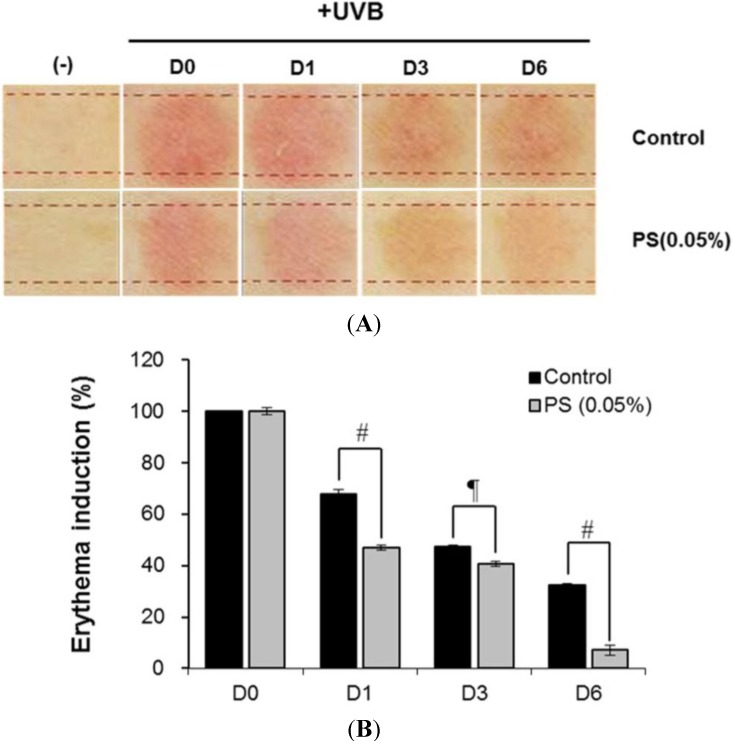
Skin recovery effects of phloretin 3',3-disulfonate were evaluated in human studies. Test areas on the forearms of 18 healthy subjects were treated with phloretin 3',3-disulfonate or the vehicle for 30 min. Subsequently, the test areas were irradiated with 1.5-fold the MED and the induced erythema was measured photometrically at the first visit (base line: before UV irradiation), 0 (after UV irradiation), 1, 3 and 6 days after application of phloretin 3',3-disulfonate. (**A**) Digital images and (**B**) induction percentage of erythema on forearm following 6 consecutive days application of phloretin 3',3-disulfonate. Data are presented as the mean ± standard deviation (*N* = 18), ^¶^ *p* < 0.05 compared with the UVB-treated group, ^#^ *p* < 0.01 compared with the UVB-treated group; PS: Phloretin 3',3-disulfonate.

The skin recovery effect of phloretin 3',3-disulfonate was also evaluated *in vivo* using the UV erythema test (Figure 8). UV erythema test was performed in 18 healthy female subjects (average age: 38.7 ± 7.6 years) with Fitzpatrick skin type I, II, III who had no history of allergenic contact dermatitis. The investigator fully explained the purpose and procedures of the study, schedule, compensation, and anticipated adverse reactions or side effects.

Test areas on the forearms of 18 healthy subjects were treated with phloretin 3',3-disulfonate or the vehicle for 30 min. Subsequently, the test areas were irradiated with 1.5-fold of the minimal erythema dose (MED) and the induced erythema was measured photometrically at the first visit (base line: before UV irradiation), 0 (after UV irradiation), 1, 2, 3 and 6 days after application of phloretin 3',3-disulfonate. The erythema index was evaluated by Mexameter^®^ MX18 (Courage + Khazaka electronic GmbH, Cologne, Germany). The phloretin 3',3-disulfonate group had significantly reduced UV-induced skin erythema at 6 days after topical application compared to the control group (Figure 8A,B); phloretin 3',3-disulfonate thus had a recovery effect on UV-induced damaged skin in a clinical trials.

### 2.8. Human Skin Primary Irritation Test of Phloretin 3',3-Disulfonate

A patch test was performed to evaluate the irritation effect of phloretin 3',3-disulfonate for clinical applications to human skin. Thirty healthy Korean subjects with Fitzpatrick skin type I, II, III were selected on the basis of inclusion and exclusion criteria, and written consent was obtained in each case. The average age was 42.2 years (range 20–49: all females). As shown in Table 2, none of the 30 subjects experienced a reaction based on the 48 and 72 h readings in our study. Specifically, we did not observe in the study subjects any adverse reactions such as erythema, burning or pruritus that were related to the topical treatment of phloretin 3',3-disulfonate.

**Table 2:** Human skin primary irritation test.

No.	Test Material	48 h					72 h					Reaction Grade ^b^		
		±	1+	2+	3+	4+	±	1+	2+	3+	4+	48 h	72 h	Mean
1	Squalene	- ^a^	-	-	-	-	-	-	-	-	-	0	0	0
2	Phloretin 3',3-disulfonate (0.1%)	-	-	-	-	-	-	-	-	-	-	0	0	0

^a^ No reaction; ^b^ Reaction grade = ∑[{Grade × No. of Responders}/{4 (Maximum grade) × 30 (Total Subjects)}] × 100 × (1/2).

## 3. Experimental Section

### 3.1. Chemicals and Antibodies

The electrophoresis reagents and protein assay kit were purchased from Invitrogen (Carlsbad, CA, USA). Antibodies against cleaved poly (ADP-ribose) polymerase (PARP), pro-caspase 3, cleaved-caspase 8, cleaved-caspase 9 and Bcl-xL were obtained from Cell Signaling Technology (Beverly, MA, USA). COX-2 and β-actin were purchased from Santa Cruz Biotechnology (Santa Cruz, CA, USA). Monochlorobimane (MCB) was purchased from Molecular Probes (Eugene, OR, USA). Propidium iodide (PI) was purchased from Sigma Chemical Co. (St. Louis, MO, USA). All other reagents were of analytical grade and purchased from Sigma.

### 3.2. Modification of Phloretin

Phloretin derivatives containing phloretin sodium sulfonate were synthesized by chemical methods (Figure 1). A weighed quantity of the phloretin (274 g) was introduced into the reaction flask and cooled in an ice-bath. The calculated quantity of concentrated sulfuric acid (5.48 L), was slowly introduced at 0 °C. After being stirred for 2 h, the reaction mixture was poured into saturated salt water through a dropping funnel with stirring and cooling in an ice-bath. After 4 h, the light red solid was precipitated, and then filtrated. The light red solid was washed with a solution of 1 M Na_2_CO_3_. The crude product was subjected to silica gel CC with a gradient of CH_2_Cl_2_-MeOH to give 3 fractions (A–C). Fraction B was target compound and recrystallized (271 g, yield: 56.7%).

### 3.3. Analysis of Phloretin and Phloretin 3',3-Disulfonate

The HPLC setup consisted of 2695 separations module (WATERS, Milford, MA, USA) and a 2996 photodiode array detector (WATERS, Milford, MA, USA). A Luna C_18_ column (150 mm × 4.6 mm, 5 μm, PHENOMENEX) was used. The samples were injected by 10 μL and eluted by isocratic runs of 20 min with a mobile phase that consisted of 10 mM sodium phosphate buffer and acetonitrile (70:30, *v*/*v*). The solvent flow rate was set at 1.0 mL/min at 25 °C. The absorbance was recorded at 285 nm.

### 3.4. Water Solubility Measurement

The water solubility of phloretin and phloretin 3',3-disulfonate were determined according to the method described by Li *et al.* [27]. Excess samples were mixed in 15 mL tubes at room temperature. An ultrasonic cleaner (BRANSON8210, Branson Ultrasonic Corporation, Danbury, CT, USA) was used to maximize the solubility of each component. After sonication at room temperature for 1 h, the sample was diluted and filtered through a 0.20 μm membrane for HPLC analysis of the sample solution concentration.

### 3.5. Cell Culture

The HaCaT human keratinocyte cell line was purchased from CLS (Eppelheim, Germany). The HaCaT cells was cultured in Dulbecco’s modified Eagle’s medium (DMEM) supplemented with 10% fetal bovine serum, 100 units/mL penicillin and 100 µg/mL streptomycin sulfate. Cells were incubated in a humidified atmosphere of 5% CO_2_ and 95% air at 37 °C.

### 3.6. UV Irradiation and Treatment

For UV irradiation experiments, the HaCaT keratinocytes were cultured on 60 mm culture dishes for 48 h. The cells were then incubated with 1% serum medium for 12 h and exposed to UVB irradiation using a LZC-UVB lamp (Luzchem, Ottawa, ON, Canada), which had an emission spectrum of 280–370 nm and a peak at 312 nm. The UV dose was measured with a UV light meter UV-340 (Lutron, Coopersburg, PA, USA). The cells were replenished with 1% serum medium including phloretin 3',3-disulfonate and followed for up to 12 h post-irradiation.

### 3.7. Cell Viability Assay

The cytotoxicity of phloretin 3',3-disulfonate after UVB irradiation was determined using 3-[4,5-dimethylthiazol-2-yl]-2,5-diphenyltetrazolium bromide (MTT) reduction to the corresponding blue formazan by viable cells. Cells were grown to ~80% confluence and maintained in 1% serum medium for 12 h prior to UV exposure. The level of blue formazan was measured spectophotometrically and used as an indirect index of cell density. Briefly, cells were exposed to MTT (1 mg/mL) for 3 h at 37 °C. The medium was removed, and the cells were solubilized with dimethyl sulfoxide. After complete solubilization, the presence of blue formazan was evaluated spectrophotometrically by measuring absorbance at 540 nm (reference, 620 nm) with an enzyme-linked immunosorbent assay (ELISA) plate reader. Viability was expressed as a percentage of the control.

### 3.8. Analysis of DNA Damage by the Comet Assay

UVB-induced DNA damage on a per cell basis was determined using the comet assay, as described previously [25]. HaCaT cells were exposed to UVB (20 mJ/cm^2^) and harvested 12 h later for the comet assay. Briefly, the cells were harvested and re-suspended in ice cold PBS after UVB treatment. Approximately 1 × 10^4^ cells in 80 µL of 0.5% (*w*/*v*) low melting point agarose were pipetted onto a frosted glass slide coated with a thin layer of 1.0% (*w*/*v*) agarose, covered with a coverslip, and placed on ice for 10 min. The cover slip was removed and the slides were immersed in ice-cold lysis solution containing 2.5 M NaCl, 10 mM Tris, 100 mM Na_2_-EDTA, and 1% (*w*/*v*) *N*-lauroyl-sarcosine, pH 10.0, and 1.0% Triton X-100 was added immediately before use. After 2 h at 4 °C, the slides were placed into a horizontal electrophoresis tank filled with buffer (0.3 M NaOH, 1 mM EDTA, pH 13) and subjected to electrophoresis for 30 min at 300 mA. The slides were transferred to neutralization solution (0.4 M Tris-HCl) for 3 × 5 min washes and stained with SYBR green I for 5 min. The comets were examined and photographed using a fluorescence microscope. Slides were viewed using the 20× objective of an Evos fluorescent microscope equipped with epifluorescence optics (Advanced Microscopy Group, Bothell, WA, USA). The tail lengths (μm) of a minimum of 50 comets in each sample were analyzed using software imaging analysis (Comet assay IV; Perceptive Instruments, Bury St. Edmunds, UK). The length of the comet was quantified as the distance from the centrum of the cell nucleus to the tip of the tail in pixel units, and tail length was expressed as mean ± standard deviation (SD) from 50 comets.

### 3.9. Cyclobutane Pyrimidine Dimer (CPD) Quantification

DNA was isolated using DNAzol reagent (Invitrogen, Carlsbad, CA, USA). DNA concentration was measured on a BioTek Epoch spectrophotometer (BioTek, Winooski, VT, USA) and the CPDs were quantified with an Oxiselect Cellular UV-induced DNA damage ELISA kit from Cell Biolabs (San Diego, CA, USA), according the manufacturer’s instructions.

### 3.10. Immunofluorescence

The cells were fixed in formaldehyde 12 h after irradiation (20 mJ/cm^2^ UVB) and stained using primary antibody (p-p53 S15; γ-H2AX) and the secondary antibody conjugated with Alexa Fluor 488 or Cy5. Nuclei were stained using Hoechst 33342, exhibiting blue fluorescence. Plates were imaged on a GE IN Cell Analyzer 1000 (GE Healthcare Ltd., Buckinghamshire, UK), and the images were analyzed with GE IN Cell Analyzer 1000 Workstation software. The number of Alexa Fluor 488 or Cy5-positive cells/100 Hoechst 33,342-positive cells was determined in 2 individual high-power fields per experiment by two independent assessors.

### 3.11. FACS Analysis

Both adherent and floating cells were collected, washed with ice-cold PBS, and fixed with 70% ice-cold ethanol overnight at 4 °C 12 h following UVB irradiation and/or afzelin treatment. Fixed cells were washed twice with PBS and treated with 100 µg/mL RNase for 30 min at 37 °C and then stained with 1 mg/mL PI in PBS containing 0.05% Nonidet-P40. The cells were then analyzed with a FACScan flow cytometer (Becton Dickinson, Franklin Lakes, NJ, USA). The percentages of cells in different cell cycle phases were evaluated from an analysis of DNA histograms. Cells with a sub-G_0_/G_1_ DNA (sub-G_1_) were considered apoptotic cells.

### 3.12. DNA Fragmentation Assay

Oligonucleosomal DNA fragmentation was identified by agarose gel electrophoresis. To determine the degradation of chromosomal DNA into nucleosome-sized fragments, a 500 μL aliquot of the lysis buffer (100 mM Tris-HCL, pH 8.5, 5 mM EDTA, 0.2 M NaCl, 0.2% SDS, and 0.2 mg/mL proteinase K) was added to the cell pellet (2 × 10^5^ cells) and incubated at 37 °C overnight. DNA was obtained by ethanol precipitation, separated on a 1.5% agarose gel, and visualized under UV light.

### 3.13. Western Blot Analysis

Cells were washed twice with cold PBS and lysed in 150 µL of sample buffer (100 mM Tris-HCl, pH 6.8, 10% glycerol, 4% SDS, 1% bromophenol blue, and 10% β-mercaptoethanol). The proteins were resolved on a NuPAGE Novex 10% Bis-Tris Gel (Invitrogen, Carlsbad, CA, USA). Following electrophoretic transfer of the proteins onto nitrocellulose membranes, they were subsequently hybridized with primary antibody (1:1000) followed by a horseradish peroxidase-conjugated secondary antibody (1:2000). Finally, the protein bands were visualized using the PowerOpti-ECL Western Blotting Detection reagent (Anigen, Hwaseong, Korea). Protein bands were quantified with Image J software (US National Institutes of Health, Bethesda, MD, USA).

### 3.14. Enzymatic Caspase Activity Assay

Activity of individual caspases in PMNs was measured by assessing cleavage of a tagged caspase-3 [(Asp-Glu-Val-Asp)-AFC (7-amino-4-trifluoromethyl-coumarin)], caspase-8 [(Ile-Glu-Thr-Asp)-AFC (7-amino-4-trifluoromethyl coumarin)], or caspase-9 [(Leu-Glu-His-Asp)-AMC (7-amino-4-methylcoumarin)] substrate peptide using ApoAlert Caspase Fluorescent Assay kits (BD Clontech, Palo Alto, CA, USA). Fluorometric detection was performed at excitation and emission wavelengths of 400 and 505 nm for caspase-3 and caspase-8, or 380 and 460 nm for caspase-9, using a fluorometer. (INFINITE M200, Tecan, Männedorf, Switzerland).

### 3.15. Real Time PCR

After each treatment, cells were harvested and homogenized in lysis buffer. Total RNAs were extracted from harvested keratinocytes using RNeasy^®^ Mini kit (Qiagen, Valencia, CA, USA), according to the manufacturer’s protocol. RNA concentration and purity was assessed using the Epoch microplate reader (BioTek, Winooski, VT, USA). First strand cDNA was synthesized from 1 to 2 µg total RNA by using the RT^2^ first strand kit (Qiagen, Valencia, CA, USA) and quantitative PCR was performed with RT^2^ qPCR Primer Assay using the manufacturer’s protocol (Qiagen, Valencia, CA, USA) and the recommended two-step cycling program, on a Takara PCR Thermal Cycler MP device (Takara, Tokyo, Japan).

Each reaction was performed in triplicate. All human qRT-PCR primers were pre-designed, validated RT^2^ qPCR primer pairs (Qiagen, Valencia, CA, USA; see below). Relative gene expression was normalized to β-actin reference gene transcription and calculated with the ΔΔC_T_ method. Primers used for qRT-PCR were pre-designed, validated RT^2^ qPCR primer pairs (Qiagen, Valencia, CA, USA) as follows: for human genes, *XPA* (PPH01524C), *XPC* (PPH01536F), *DDB2* (PPH01726A), *RPA1* (catalog no. PPH02730A) and *ACTB* (β-actin, PPH00073G).

### 3.16. Intracellullar GSH Level

Intracellular GSH levels were determined by using a GSH-sensitive fluorescence dye monochlorobimane (MCB). HaCaT cells (1 × 10^6^ cells/mL) were incubated with 5 μM MCB cell tracker for 30 min. Intensity of MCB cell tracker fluorescence by GSH was analyzed by the INFINITE M200 Fluoremeter (Tecan, Männedorf, Switzerland) at fluorescence DAPI region (excitation, 351 nm; emission, 380 nm).

### 3.17. Inflammatory Cytokine Assay

The cells were irradiated with the indicated UVB doses and then incubated with the indicated phloretin 3',3-disulfonate concentration for 12 h. After 12 h, PGE_2_ and IL-6 concentrations in the culture supernatant were measured by ELISA kits (R&D Systems, Minneapolis, MN, USA), according to the manufacturer’s instructions.

### 3.18. Clinical Study of Skin Recovery Effect of Phloretin 3',3-Disulfonate on UV-Induced Damage Skin

Eighteen female subjects (average age: 38.7 ± 7.6 years) with Fitzpatrick skin types I, II, III participated in this study. The erythema was induced on the forearms with 1.5 MED of individual subjects by Multiport UV Solar Simulator (SOLAR LIGHT CO., Philadelphia, PA, USA). The 2 test sites (including non-treatment site) were UV irradiated. At 0 day (after UV irradiation), 1, 2, 3 and 6 days after application of the test product (Phloretin 3',3-disulfonate 0.05%), clinical scoring of erythema was assessed by two researchers according to a 6-scale scoring method (0–4 grade). The erythema index was evaluated using a Mexameter^®^ MX18 (Courage + Khazaka electronic GmbH, Cologne, Germany). Erythema index on the test sites (UV-induced damage area on a forearm) were evaluated thrice before treatment (base line: before UV irradiation), and at 0 day (after UV irradiation), 1, 2, 3 and 6 days after application. This study was approved by the ethics committee of the DERMAPRO/Skin Reseach Center (Seoul, Korea), subjects gave written informed consent.

### 3.19. Human Skin Primary Irritation Test

Thirty healthy Korean subjects with Fitzpatrick skin type I, II, III were selected on the basis of inclusion and exclusion criteria, and written consent was obtained in each case. The average age was 42.2 years (range 20–49: all females). The subjects had no history of allergic contact dermatitis, nor had they used topical or systemic irritant preparations in the previous month. Phloretin 3',3-disulfonate (0.1%) formulated with squalene was prepared and applied. The patches (chambers) stayed in place for 48 h. Once the patches were removed, a reading was done 48 and the 72 h later; readings were scored according to a modified criteria proposed by Frosch and Kligman [28] and the Cosmetic, Toiletry, and Fragrance Association (CTFA) guidelines [29] as follows: 0 = no reaction; 1 = slight erythema, spotty of diffuse; 2 = moderate uniform erythema; 3 = intense erythema with erythema; 4 = intense erythema with edema and vesicles. This study was approved by the ethics committee of the DERMAPRO/Skin Reseach Center, and subjects gave written informed consent.

### 3.20. Statistical Analysis

All data were expressed as mean ± SD. Differences between the control and treatment group were evaluated by Student’s *t-*test using the Statview software (Abacus Concepts, Piscataway, NJ, USA). A *p* < 0.01 was considered statistically significant [30].

## 4. Conclusions

In conclusion, the data acquired in this study demonstrated that modified phloretin, phloretin 3',3-disulfonate, had more potential photo-protective properties than phloretin, while the solubility of phloretin 3',3-disulfonate improved greatly as compared to phloretin Additionally, phloretin 3',3-disulfonate reduced UV induced skin injuries (DNA damage, redox state imbalance and inflammation), and was demonstrated to be safe to use on humans. Phloretin 3',3-disulfonate has potential as an effective therapeutic agent providing protection against UVB-induced skin disorders.

## Supplementary Materials

Supplementary materails can be found at http://www.mdpi.com/1422-0067/15/10/18919/s1.

## Supplementary Files

Supplementary File 1
