# Supplementary material for: Protective Effects of a New Phloretin Derivative against UVB-Induced Damage in Skin Cell Model and Human Volunteers
Source: Int J Mol Sci. 2014 Oct 20;15(10):18919–40. doi: 10.3390/ijms151018919 (PMC4227253; doi:10.3390/ijms151018919)

Supplementary Information

Figure S1. UVB irradiation induced p53 (Ser-15) and γ-H2AX phosphorylation in a dose-dependent manner in HaCaT keratinocytes. Cells were irradiated with various concentrations of UVB (0–20 mJ/cm^2) for 12 h after being exposed to UVB. At 12 h after UVB irradiation, cells (2 × 10^4 cell/100 µL) were fixed/stained to measure the levels of phosphoactive histone H2A.X (γH2A.X; green) and phospho-p53 (serine 15) (red). Staining with Hoechst 33342 (blue) was performed to observe cell nuclei. Cells were imaged on the GE IN Cell Analyzer 1000 at 20× objective magnification. Scar bar = 200 µm. Data are mean ± standard deviation of 3 independent experiments. ¶ $p < 0.05$ compared with the unirradiated group, # $p < 0.01$ compared with the unirradiated group.

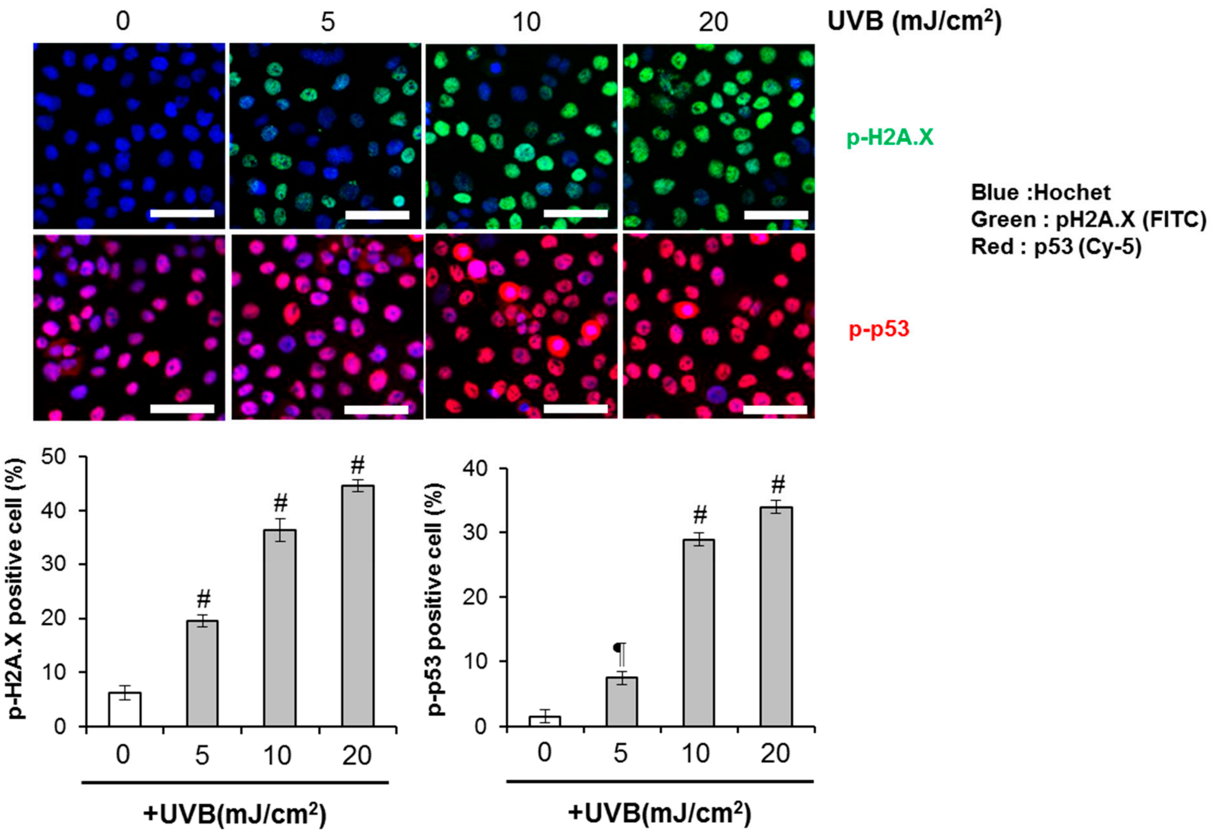

Supplement: Supplementary File 1 [file ijms-15-18919-s001.pdf]
